# Long-term experience with enzyme replacement therapy (ERT) in MPS II patients with a severe phenotype: an international case series

**DOI:** 10.1007/s10545-014-9686-7

**Published:** 2014-03-05

**Authors:** Christina Lampe, Ann-Kathrin Bosserhoff, Barbara K. Burton, Roberto Giugliani, Carolina F. de Souza, Camila Bittar, Nicole Muschol, Rebecca Olson, Nancy J. Mendelsohn

**Affiliations:** 1 Department of Pediatric and Adolescent Medicine, Villa Metabolica, University Medical Center of Mainz, Mainz, Germany; 2 Ann and Robert H. Lurie Children’s Hospital and Northwestern University Feinberg School of Medicine, Chicago, IL USA; 3 Medical Genetics Service/Hospital de Clinicas de Porto Alegre (HCPA), Porto Alegre, RS Brazil; 4 Department of Genetics, Universidade Federal do Rio Grande do Sul, Porto Alegre, RS Brazil; 5 National Institute of Population Medical Genetics (INAGEMP), Porto Alegre, RS Brazil; 6 Department of Pediatrics, University Medical Center Hamburg-Eppendorf, Hamburg, Germany; 7 Department of Medical Genetics, Children’s Hospitals & Clinics of Minnesota, Minneapolis, MN USA; 8 Division of Genetics, Department of Pediatrics, University of Minnesota, Minneapolis, MN USA; 9 Department for Pediatric and Adolescent Medicine, Dr Horst Schmidt Clinics (HSK), Ludwig-Erhard-Strasse 100, 65199 Wiesbaden, Germany

## Abstract

**Introduction:**

No published clinical trial data are available to inform the use of enzyme replacement therapy (ERT) in patients with the severe (neuropathic) phenotype of mucopolysaccharidosis II (MPS II). Current guidelines recommend ERT administered intravenously be used on a trial basis in this population.

**Aims/methods:**

A retrospective chart review was conducted at five international centers for this case series of 22 patients with neuropathic MPS II who received intravenous idursulfase 0.5 mg/kg weekly for at least 2 consecutive years. We collected data about urinary glycosaminoglycan levels, adverse events, and the following somatic signs/symptoms: skeletal disease, joint range of motion, liver/spleen size, respiratory infections, cardiac disease, diarrhea, skin/hair texture, and hospitalizations.

**Results:**

The age at diagnosis was 2 months to 5 years, and the age at idursulfase initiation was between 18 months and 21 years. One of 22 patients experienced improvements in seven somatic signs/symptoms; 17/22 experienced improvements in five to six somatic signs/symptoms; and 4/22 experienced improvements in four somatic signs/symptoms. None experienced fewer than four improvements. No new safety concerns arose. Infusion-related reactions were experienced by 4/22 patients but were successfully managed using accepted strategies.

**Conclusions:**

Long-term treatment with idursulfase was associated with improvements in somatic manifestations in this case series of patients with neuropathic MPS II. The family and medical team should maintain open lines of communication to make treatment decisions that take into consideration the benefits and limitations of ERT in this population.

**Electronic supplementary material:**

The online version of this article (doi:10.1007/s10545-014-9686-7) contains supplementary material, which is available to authorized users.

## Introduction

Mucopolysaccharidosis II (MPS II, Hunter syndrome, OMIM 309900) is an X-linked lysosomal storage disorder with an incidence of 0.3–0.71 per 100,000 live births (Bach et al 1973; Burton and Giugliani 2012). MPS II is caused by a deficiency in the enzyme iduronate-2 sulfatase (I2S, EC 3.1.6.13), leading to the accumulation of the glycosaminoglycans (GAGs) dermatan sulfate and heparan sulfate in lysosomes. Excessive storage of these GAGs causes a variety of clinical manifestations, including coarse facies, hearing loss, cardiac valve disease, restrictive and obstructive airway disease, recurrent upper respiratory infections, hepatosplenomegaly, skeletal abnormalities, joint contractures, short stature, and a characteristic skin rash (Martin et al 2008; Neufeld and Muenzer 2001).

MPS II is a progressive disease that presents a high burden of morbidity and a reduced life expectancy (Martin et al 2008; Neufeld and Muenzer 2001). The disorder manifests on a spectrum of severity from attenuated to severe. Approximately one-third of patients have attenuated disease, with a gradual onset, lack of cognitive involvement, and life expectancy into the fourth through sixth decade of life (Young and Harper 1982; Young et al 1982a). The remaining two-thirds have severe disease characterized by the onset of signs and symptoms before the age of 3 years, progressive cognitive impairment, and behavioral disturbances. Natural history data from untreated severe patients indicate a life expectancy only into the second or third decade (Young and Harper 1983; Young et al 1982a, b). Of note, disease severity does not refer to the extent or seriousness of somatic signs and symptoms but only to the rate of disease progression and the presence or lack of cognitive involvement. All patients, regardless of disease severity, experience similar somatic manifestations that reduce the quality of life of both patients and caregivers (Martin et al 2008).

Idursulfase (Elaprase®, Shire Human Genetic Therapies, Inc., Lexington, MA, USA), a recombinant human I2S enzyme replacement therapy (ERT), was approved in the United States in 2006 and in Europe in 2007 for the treatment of MPS II. It is currently available in over 50 countries. The approval of idursulfase was based on a pivotal phase II/III study which enrolled 96 patients between the ages of 5 and 31 years (Muenzer et al 2006). Patients were randomized to receive weekly or every-other-week infusions of idursulfase 0.5 mg/kg or placebo infusions for 53 weeks. The primary efficacy endpoint was a composite of distance walked in 6 min (6MWT) and improvements in percent predicted forced vital capacity (%FVC). The study found that patients in both ERT groups exhibited significant improvements in the primary composite endpoint compared with those in the placebo group, with the greatest gains seen in the weekly treatment group (Muenzer et al 2006). All 94 patients who completed the placebo-controlled study were enrolled into an open-label extension trial in which they received weekly infusions of 0.5 mg/kg idursulfase for an additional 2 years. Patients experienced improvements in absolute FVC, 6MWT distance, liver and spleen volumes, shoulder range of motion, and parent- and child-assessed Child Health Assessment Questionnaire Disability Index Scores (Muenzer et al 2011).

Because of the difficulties involved in collecting FVC and 6MWT data from cognitively impaired patients, the pivotal trial and its extension enrolled only individuals with attenuated phenotypes. While there are no clinical trial data available for severe patients, clinical experience suggests that severe patients can experience certain somatic improvements and caregiver-reported improvements in quality of life. In a recent consensus report, a panel of experts described their experience with idursulfase in 66 patients with the severe phenotype (Muenzer et al 2012). After at least 1 year of ERT, 50 of these patients experienced at least one type of somatic improvement. These improvements included reductions in the frequency of respiratory infections, reductions in liver volume and sleep apnea, and improvements in joint range of motion. In 61 out of 66 cases, physicians and families found sufficient benefit to continue ERT. Current U.S. and European guidelines suggest initiating a 6- to 18-month trial of ERT in severe MPS II patients to assess the response before stopping or continuing therapy (Muenzer et al 2012; Scarpa et al 2011). Here we describe our experiences with long-term (≥ 2 years) ERT in a series of severe MPS II patients from five international centers.

## Methods

A retrospective chart review was conducted at the authors’ institutions to identify all MPS II patients who were treated with intravenous (IV) idursulfase according to the prescribing information (0.5 mg/kg once weekly) continuously for at least 24 consecutive months. All patients had neuropathic MPS II as confirmed by formal developmental testing or by investigator report.

The following data were collected from the patients’ records:

- Liver and/or spleen size as measured via palpation and/or imaging studies.
- Frequency of respiratory infections as reported by caregivers.
- Texture of hair/skin as observed during physical examination.
- Frequency of diarrhea as reported by caregivers.
- Joint range of motion as observed during physical examination.
- Skeletal disease as measured via imaging studies.
- Cardiac disease as measured via echocardiogram and/or electrocardiogram.
- Urinary glycosaminoglycan (uGAG) levels as assessed by chart review.
- Disease-related hospitalizations as assessed by chart review.
- Cognitive function as assessed by formal developmental evaluation and/or by investigator impression if formal assessments were not performed.

Testing for IgG and IgE antibodies to idursulfase, when reported, was conducted by the Bioanalytical and Biomarker Development group, Research and Development, Shire, Lexington, MA, USA.

## Results

### Patient demographics

Patients received ERT at five international centers. In Brazil, three patients received ERT at one center. In Germany, 12 patients received ERT at two centers, and in the United States, seven patients received ERT in two centers. The mean age at diagnosis was 2.8 years (range: 0.2–5.0 years). The mean age at the start of ERT was 6.8 years (range: 1.5–21.0 years), and the mean duration of therapy was 4.7 years (range: 2.0–6.0 years). Individual patient demographics are presented in Supplementary Table 1.

### Safety

Four out of 22 (18 %) patients experienced at least one infusion-related reaction (IRR) at any time during the course of ERT (Supplementary Table 1). No other adverse events were observed.

Prophylactic medications to prevent IRRs were administered to 10/22 (45 %) patients. Of these, four patients had experienced previous IRRs. The remaining six patients were administered prophylactic medications without a history of IRRs, and none of them developed reactions. Of the ten patients who received prophylactic medications, seven patients received antihistamines alone, one of whom had experienced a previous IRR. The remaining three patients received antihistamines plus steroids; all of these patients had experienced prior IRRs.

Of the seven patients who were treated with antihistamines, two were weaned off all prophylactic medication after approximately 1 year. Neither of these patients had experienced an IRR. Of the three patients who were treated with antihistamines plus steroids, two were weaned to antihistamines alone after approximately 1 year but continued to remain free of subsequent IRRs.

Seventeen patients were tested for IgG and IgE anti-drug antibodies (ADA). Of these patients, six (35 %) developed IgG ADAs, and none developed IgE ADAs. No clear correlation between ADA positivity and IRRs was observed. Only two of the six ADA-positive patients experienced IRRs (Table 1).

**Table 1:** Anti-drug antibody and infusion-related reaction status among cases tested (*n* = 17)

	IRR positive	IRR negative
ADA positive	2	4
ADA negative	3	8

*ADA* anti-drug antibodies, *IRR* infusion-related reaction

### Response to treatment

According to our observations, all of the patients experienced somatic improvements on ERT (Table 2). The majority of the patients (18 patients, 82 %) had improvements in five to seven signs and symptoms, and all of the patients experienced improvements in at least four of the somatic signs and symptoms evaluated. Of the signs and symptoms evaluated (Table 3), all patients experienced reductions in liver and/or spleen size (as seen on imaging studies or physical examination), reduced frequency of respiratory infections (as recorded in patient charts), and improvements in hair and/or skin texture (as judged by the investigator). Eleven out of 14 (79 %) patients who had been hospitalized for a MPS II-related reason prior to starting ERT showed a reduction in the number of disease-related hospitalizations on ERT as recorded in the patients’ charts. Improvement in joint range of motion was reported for 7/21 patients (33 %), and another 13/21 patients (62 %) showed stabilization of joint disease. Note that measurement techniques were not standardized among centers; however, an increase in joint range of motion of at least 10° was considered an improvement. Skeletal disease, as assessed by imaging studies, was stabilized in 19/22 (86 %) patients. Cardiac disease, as assessed by electrocardiogram and/or echocardiogram, was also stabilized in 19/22 (86 %) patients. Stabilization can be considered a positive response to treatment in a progressive disease like MPS II. Interestingly, one patient (patient 4) experienced partially improved cardiac disease on treatment with idursulfase. His echocardiograms prior to treatment revealed mild left ventricular dysfunction and abnormal septal motion. On treatment, he consistently had normal left ventricular systolic function. He also had evidence of mild thickening of the mitral valve, which has been stable on 6 years of treatment.

**Table 2:** Number of patients with somatic signs or symptoms assessed as “improved” on enzyme replacement therapy

Number of signs/symptoms improved on ERT	Number of patients (*n* = 22)
7	1
6	4
5	13
4	4
Fewer than 4	0

*ERT* enzyme replacement therapy

**Table 3:** Somatic response on enzyme replacement therapy

Sign/symptom	Improvement	Stabilization	Disease progression
Liver and/or spleen size^a,b^	22/22 (100 %)	0	0
Frequency of respiratory infections^c^	22/22 (100 %)	0	0
Texture of hair and/or skin^a^	21/21 (100 %)	0	0
Disease-related hospitalizations	11/14 (79 %)	3/14 (21 %)	0
Diarrhea^c^	4/9 (44 %)	5/9 (56 %)	0
Joint range of motion (1 or more joints)^a^	7/21 (33 %)	13/21 (62 %)	1/21 (5 %)
Skeletal disease^b^	2/22 (9 %)	19/22 (86 %)	1/22 (5 %)
Cardiac disease^d^	1/22 (5 %)	19/22 (86 %)	2/22 (9 %)

^a^Assessed by physical exam

^b^Assessed by imaging studies

^c^Assessed by caregiver report

^d^Assessed by electrocardiogram and/or echocardiogram

**Fig. 1:**
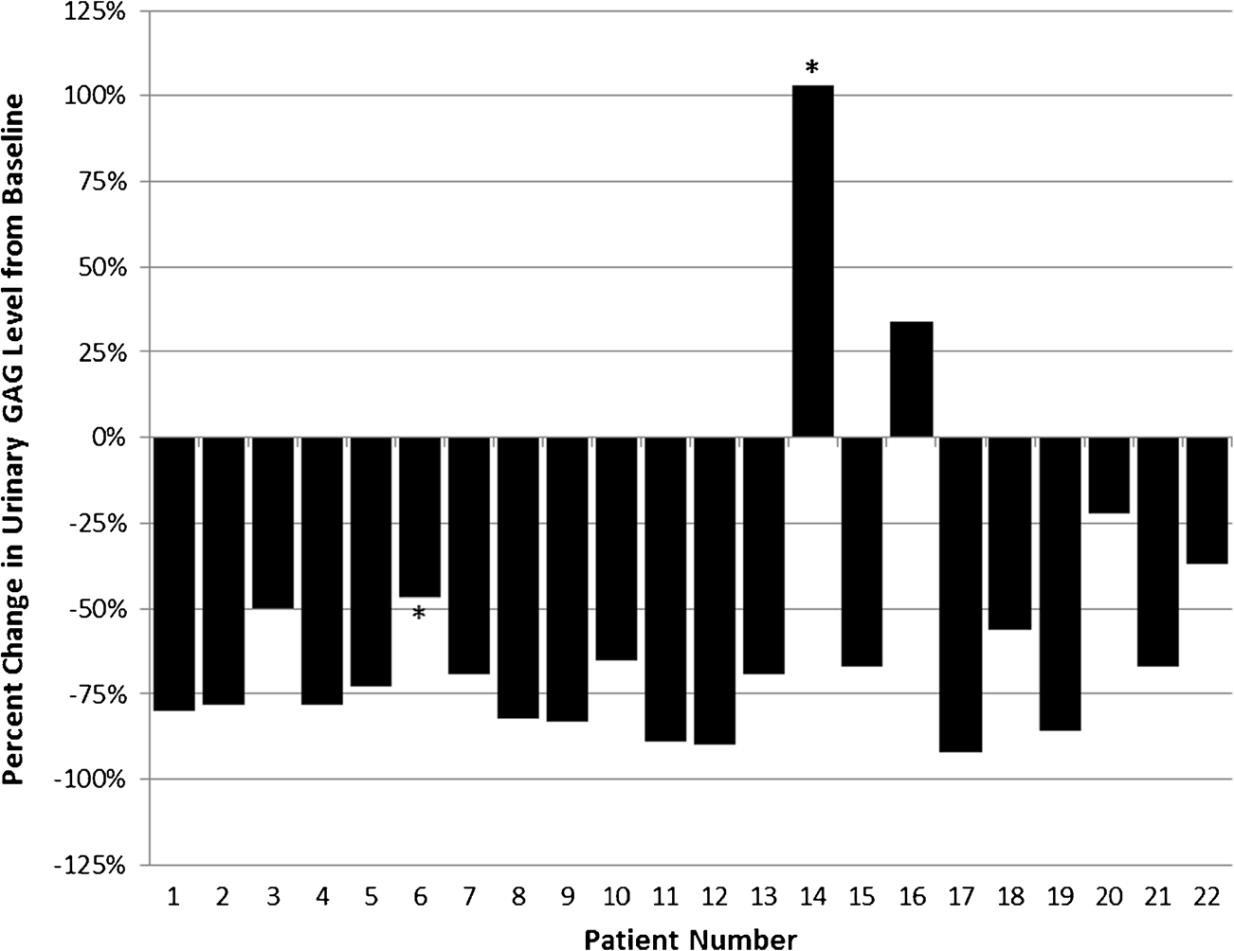
Percent change in urinary glycosaminoglycan (uGAG) levels from baseline at last available test result for severe MPS II patients on long-term enzyme replacement therapy (ERT). *First available uGAG test result for patient 6 was after 7 months of ERT and for patient 14 was after 6 months of ERT. No baseline data are available. Here we report percent change between first available and last available uGAG test results

Urinary GAG levels were tested, and individual changes in uGAG levels are detailed in Fig. 1. A decrease in uGAG levels compared with baseline was observed in 20/22 (91 %) patients, with decreases ranging from 22 to 97 %. Of note, two patients (patients 6 and 14) did not have baseline test results available; we therefore calculated the percent change in uGAG level between the first available test result and the last available test result. Of the 22 patients, two demonstrated an increase in uGAG levels. Patient 16 was ADA-negative but did experience IRRs. His baseline uGAG value was 10.14 mg/mmol creatinine (upper limit of normal (ULN): 8.30 mg/mmol creatinine). This level rose to a high of 23.52 mg/mmol creatinine at approximately 6 months after the start of ERT. At the last available measurement 3 years after the start of ERT, the level had fallen to 13.58 mg/mmol creatinine. Despite the fluctuations in uGAG level, he experienced improvements in liver and spleen size, frequency of respiratory infections, hair and skin texture, and the number of disease-related hospitalizations on ERT. His joint range of motion, skeletal disease, and cardiac disease stabilized on ERT. The second patient, patient 14, was ADA-positive and had no IRRs. His case is complicated by a lack of a true baseline measurement. His uGAG levels were nearly normal at 7.86 mg/mmol creatinine at 6 months on ERT (ULN: 7.70 mg/mmol creatinine), then rose to a high of 25.09 mg/mmol creatinine 2 years after the start of ERT. At the last available measurement, 4 years after the start of ERT, his uGAG levels were 15.93 mg/mmol creatinine. Despite elevated uGAG levels, this patient experienced improvements in liver and spleen size, frequency of respiratory infections, hair and skin texture, and the number of disease-related hospitalizations. His skeletal and cardiac disease parameters remained stable on ERT, although his joint range of motion limitations progressed.

Cognitive disease was assessed by formal developmental evaluation or by investigator impression when formal testing was not performed. In this case series, cognitive disease progressed in 17/22 (77 %) patients and stabilized in 3/22 (14 %). Slight improvements were reported for 2/22 (9 %) patients, both of whom were evaluated by investigator impression and not by formal developmental testing. Because idursulfase does not cross the blood–brain barrier (Boado et al 2013), these improvements are likely due to improved sleep or respiration leading to the children having greater interactions with their environments and are not due to any direct action of the drug upon the brain.

## Discussion

Because clinical trial data for the use of idursulfase to treat severe (neuropathic) MPS II are lacking, there has been much interest in the long-term clinical course of such patients. In a recent consensus report, a panel of experts pooled their experiences with treating 66 severe patients with ERT and concluded that somatic signs and symptoms were improved in the majority. The panel recommended that a trial of 6 to 12 months’ treatment be offered to severe patients (Muenzer et al 2012). Follow-up examinations are recommended at 6- to 12-month intervals during the trial of treatment (Scarpa et al 2011). In the present case series, the long-term effects of ERT with idursulfase were analyzed in 22 patients with severe MPS II who received ERT for at least 2 consecutive years at five international centers. In agreement with the expert consensus panel, we found that all of our patients experienced improvements in at least four of the somatic signs and symptoms evaluated, and 82 % experienced improvements in five to seven somatic signs and symptoms (Table 2).

Long-term treatment with idursulfase was generally well-tolerated in this case series. The only reported adverse events were IRRs, with 4/22 (18 %) patients experiencing manageable reactions. These included extreme irritability, hypertension, allergic conjunctivitis, urticaria factita, cough, tachypnea, shivering, and vomiting. All resolved with standard care. We did not find a clear connection between IRRs and ADA positivity (Table 1). All four patients who experienced IRRs were successfully premedicated before subsequent infusion visits with prophylactic antihistamines (*n* = 1) or antihistamines plus steroids (*n* = 3). Two patients in the latter group were later weaned to antihistamines alone with no further IRRs.

As an MPS II expert consensus panel has previously pointed out, a major challenge in evaluating the response to ERT in severe MPS II patients is the lack of assessment tools that are validated in this population (Muenzer et al 2012). Cognitive decline and behavioral difficulties decrease quality of life for patients with MPS II and their caregivers (Bax and Colville 1995), but it is clear that IV idursulfase treatment does not address these disease features (Muenzer et al 2012; Wraith et al 2008) because the large protein does not appear to cross the blood–brain barrier (Boado et al 2013). Like Muenzer and colleagues (Muenzer et al 2012), we do consider, however, that improvements in somatic signs and symptoms may be experienced by severe patients and can help improve the quality of patients’ and caregivers’ lives. For example, frequent respiratory infections leading to hospitalization are a common finding in MPS II, causing distress for patients and caregivers as well as lost time at work for caregivers (Muhlebach et al 2011; Young et al 1982a). In the current case series, 100 % of patients experienced a reduction in the frequency of respiratory infections with ERT, and 79 % had reductions in the number of disease-related hospitalizations as judged by the managing physician who reviewed patient records (Table 3). In our experience, reductions in organomegaly can improve breathing and reduce abdominal pain, nausea, and early satiety among MPS II patients. All of our patients in this case series experienced reductions in liver and/or spleen size on ERT. Joint contractures and skeletal deformities associated with dysostosis multiplex result in significant restrictions in mobility and progressive disability in MPS II (Link et al 2010; Wraith et al 2008). In the current study, joint range of motion improved in 33 % of patients, and stabilized in 62 % of patients. In addition, 86 % of patients showed stabilization of skeletal disease, which may be considered a benefit of treatment for a progressive disease such as MPS II. It has been previously stated that an important goal of therapy is the improvement in quality of life for the patient and family, so a perception by the family of improved quality of life should be taken into strong consideration when deciding whether or not to continue therapy (Muenzer et al 2012). We agree with this approach and suggest not prohibiting cognitively disabled patients from trying a treatment that may produce clinically relevant improvements in multiple somatic features, thereby lessening the burden of illness for the patient and family.

Urinary GAGs are a useful endpoint in order to observe the biochemical effects of idursulfase treatment. Urinary GAG levels were generally reduced on ERT, with 91 % of patients experiencing a decrease in uGAG levels as compared with baseline (Fig. 1). Two patients experienced an increase in uGAG levels while receiving idursulfase, but this increase did not clearly correspond with ADA, as patient 14 was ADA positive and patient 16 was ADA negative. Both patients experienced four or more somatic improvements on ERT despite increased uGAG levels. It is possible that these patients’ measurements may have been misleading, as individual uGAG measurements vary from day to day and even at different times during the day. The picture is somewhat more complicated for patient 14, who lacks baseline data and whose earliest uGAG test was performed after 6 months of ERT. The result at that point was nearly normal (7.86 mg/mmol creatinine; ULN: 7.70 mg/mmol creatinine). Thus, data from patient 14 should be considered with caution.

As expected for severe patients, cognitive disease progressed in 17/22 (77 %) patients and stabilized in 3/22 (14 %), as assessed by formal developmental evaluation or by investigator impression when formal evaluations were not performed. Slight improvements were reported for two patients. This likely reflects an increased ability of these patients to interact with others and with their environment due to improved mobility, improved sleep, and/or reductions in respiratory disease burden, since idursulfase does not cross the blood–brain barrier (Boado et al 2013). In our experience, families of severe MPS II patients often choose to initiate ERT despite the presence of cognitive impairment because they believe that somatic improvements will improve the quality of the patient’s life. This finding is in keeping with a published survey of MPS families, which found that 77 % of respondents were in favor of starting ERT in a patient with a severe phenotype, even knowing that treatment cannot alter the intellectual deterioration associated with the disease (Coman et al 2008).

In summary, this retrospective case series provides details on our experiences with treating severe MPS II patients with ERT for more than 2 years continuously. Treatment was associated with improvements in somatic signs and symptoms of the disease for all patients, and it was generally well tolerated. While ERT may provide benefits for severe MPS II patients, families should make any treatment decisions in concert with their child’s physician, with open lines of communication regarding the possible benefits and limitations of this treatment (Muenzer et al 2012; Scarpa et al 2011; Wraith et al 2008). Criteria for discontinuation of ERT should be thoroughly outlined before the start of therapy and evaluated again after 6 to 12 months on treatment and every 18 to 24 months thereafter.

## Electronic supplementary material

Below is the link to the electronic supplementary material.

Supplementary Table 1 (DOCX 26.4 kb)

## Abbreviations

%FVC: Percent predicted forced vital capacity
6MWT: 6 minute walk test
ADA: Anti-drug antibodies
ERT: Enzyme replacement therapy
FVC: Forced vital capacity
GAG: Glycosaminoglycan
I2S: Iduronate-2-sulfatase
IRR: Infusion-related reaction
IV: Intravenous
MPS: Mucopolysaccharidosis
uGAG: Urinary glycosaminoglycan
ULN: Upper limit of normal
