# Supplementary material for: Long-term experience with enzyme replacement therapy (ERT) in MPS II patients with a severe phenotype: an international case series
Source: J Inherit Metab Dis. 2014 Mar 5;37(5):823–9. doi: 10.1007/s10545-014-9686-7 (PMC4158409; doi:10.1007/s10545-014-9686-7)
Supplement: Supplementary file 1 — (DOCX 26.4 kb) [file 10545_2014_9686_MOESM1_ESM.docx]

|  |  |  |  |  |  | Response to Treatment | | | | | | | | | |
| --- | --- | --- | --- | --- | --- | --- | --- | --- | --- | --- | --- | --- | --- | --- | --- |
| Pt # | Age at DX (years) | Age at ERT Start (years) | Time on ERT | Infusion Reaction | Anti-drug Antibodies | uGAG | Liver and/or Spleen Size | Respiratory Infections | Cardiac Disease | Hair and/or Skin | Joint Range of Motion | Skeletal Disease | Diarrhoea | Disease-related Hospital-izations | Cognitive Involvement* |
|  |  |  |  |  |  |  |  |  |  |  |  |  |  |  |  |
| 1 | 3 | 21 | 6 yr 0 mo | no | ND | I | I | I | S | I | I | P | NA | NA | P |
| 2 | 2 | 3 | 5 yr 9 mo | no | yes | I | I | I | S | I | S | S | S | I | P |
| 3 | 1 | 12 | 5 yr 9 mo | no | yes | I | I | I | S | I | S | S | S | I | P |
| 4 | 3 | 6.5 | 5 yr 9 mo | yes | yes | I | I | I | I | I | S | S | I | NA | S |
| 5 | 3.5 | 6 | 5 yr 8 mo | yes | no | I | I | I | S | I | S | S | S | I | P |
| 6 | <1 (2 mo) | 2.5 | 5 yr 8 mo | no | no | I | I | I | S | I | S | S | NA | S | P |
| 7 | 4 | 13.5 | 5 yr 7 mo | no | yes | I | I | I | S | I | S | S | NA | I | P |
| 8 | 2 | 11.5 | 5 yr 7 mo | no | no | I | I | I | S | I | S | S | NA | NA | P |
| 9 | 4.5 | 7 | 5 yr 7 mo | yes | no | I | I | I | P | I | S | S | NA | S | P |
| 10 | 3 | 6 | 5 yr 6 mo | no | no | I | I | I | S | I | S | S | S | I | P |
| 11 | 5 | 6 | 5 yr 4 mo | no | no | I | I | I | S | I | I | S | NA | NA | P |
| 12 | 2 | 5 | 5 yr 4 mo | no | no | I | I | I | S | I | I | S | NA | NA | slight I |
| 13 | 1.5 | 3 | 5 yr 4 mo | no | no | I | I | I | S | I | I | S | NA | NA | S |
| 14 | 2 | 7.5 | 5 yr 3 mo | no | yes | P | I | I | S | I | P | S | NA | I | P |
| 15 | 4.5 | 5 | 5 yr 2 mo | no | no | I | I | I | S | I | S | S | NA | I | P |
| 16 | 4 | 4.5 | 5 yr 1 mo | yes | no | P | I | I | S | I | S | S | S | I | P |
| 17 | 3.5 | 4.5 | 3 yr 0 mo | no | yes | I | I | I | S | I | S | S | I | NA | P |
| 18 | < 1 (10 mo) | 1.5 | 2 yr 7 mo | no | ND | I | I | I | S | I | I | I | NA | I | slight I |
| 19 | 2.5 | 4.5 | 2 yr 5 mo | no | no | I | I | I | P | I | S | I | I | S | P |
| 20 | 2.5 | 8 | 2 yr 5 mo | no | ND | I | I | I | S | I | I | S | I | NA | P |
| 21 | 4 | 5 | 2 yr 5 mo | no | ND | I | I | I | S | ND | ND | S | NA | I | S |
| 22 | 4 | 6 | 2 yr 0 mo | no | ND | I | I | I | S | I | I | S | NA | I | P |

**Supplementary Table 1**. Patient demographics and individual response to enzyme replacement therapy. I, improvement; S, stabilisation; P, progression of disease; NA, not applicable; ND, no data; DX, diagnosis; Pt, patient; uGAG, urinary glycosaminoglycan level. *Cognitive impairment was assessed by formal developmental testing or by physician assessment on examination when formal developmental testing was not performed.
